# Efficacy of adjusted weight-based dosing of desmopressin (1-deamino-8-d-arginine vasopressin in type 1 von Willebrand disease

**DOI:** 10.1097/MBC.0000000000001251

**Published:** 2023-10-13

**Authors:** Craig D. Seaman

**Affiliations:** a Department of Medicine, Division of Hematology/Oncology, University of Pittsburgh; b Hemophilia Center of Western Pennsylvania, Pittsburgh, Pennsylvania, USA

## Introduction

Von Willebrand disease (VWD), the most common inherited bleeding disorder, is a heterogeneous condition caused by a deficiency or defect in von Willebrand factor (VWF). A disorder of primary hemostasis, VWD largely manifests as mucocutaneous bleeding and bleeding following hemostatic stressors, such as surgery and childbirth [1]. Desmopressin (1-deamino-8-d-arginine vasopressin) (DDAVP) is commonly used for the treatment and prevention of bleeding in VWD. It acts by stimulating the release of endogenous VWF from endothelial stores. DDAVP use is primarily restricted to patients with type 1 VWD due to ineffectiveness in most other types of VWD. Tachyphylaxis occurs with repeated use over short intervals; thus, DDAVP is ideal for the treatment of minor bleeding and prevention of bleeding in minor invasive procedures where prolonged therapy is not necessary [2].

The standard intravenous dose of DDAVP is 0.3 mcg/kg of actual body weight (ABW) [3]. This dose was established more than 40 years ago by the first randomized control trial evaluating the efficacy of DDAVP in patients with bleeding disorders undergoing dental extractions and based on a dose-response relationship that resulted in maximal factor VIII activity [4]. Whether or not this dose is appropriate in extremes of weight, such as obesity, is uncertain. In high doses used in VWD, DDAVP demonstrates minimal tissue distribution, approximating plasma volume. Given this, and its intravascular site of action, DDAVP should be effective using adjusted weight-based (ADJ) dosing, such as ideal body weight (IBW) [5]. Unfortunately, there is little published literature on this topic [6]. Despite these uncertainties, in practice, the maximum dose of DDAVP in the United States is often capped at 20–30 mcg, which is a method of using ADJ dosing [7]. With little data to guide hematologists when determining the DDAVP dose for obese patients with VWD, it is critical to determine if ADJ dosing of DDAVP is effective in this population. If ADJ dosing provides adequate hemostasis, the use of ABW, and unnecessarily high doses of DDAVP, may increase the risk of serious adverse effects, such as thrombosis and hyponatremia, and should be avoided.

## Methods

In this study, we performed a single center retrospective electronic health record (EHR) review to determine the efficacy of ADJ dosing of DDAVP in patients with type 1 VWD. Patients aged 18 or older with type 1 VWD administered DDAVP intravenously between January 1, 2020, and June 30, 2022, to treat an acute bleed or prevent periprocedural bleeding were included in the study. VWD diagnosis was determined using the International Classification for Diseases, 10th Revision (ICD-10) code D68.0 followed by review of the EHR to determine VWD type. A documented weight within 7 days of DDAVP administration was required for inclusion in the study. Exclusion criteria consisted of concomitant hereditary bleeding disorder; concomitant use of antifibrinolytics or VWF concentrate; or expected DDAVP dose less than 20 mcg based on ABW, which is often the threshold where ADJ dosing becomes a consideration.

## Results

Comparisons were performed between ADJ (expected dose of DDAVP based on ABW greater than the DDAVP dose administered) and non-ADJ (expected dose of DDAVP based on ABW equivalent to the DDAVP dose administered) dosing groups. Data collected included demographics (age, race, ethnicity, sex); body mass index (BMI), including BMI category; DDAVP dosing information; and clinical and medical conditions that may affect bleeding risk (Table 1). DDAVP dosing information consisted of expected dose based on ABW and actual dose administered. The reason for DDAVP administration, acute bleed or invasive procedure, and type of each, was recorded. Outcomes consisted of bleeding treatment efficacy and presence of periprocedural bleeding. Bleeding treatment efficacy was categorized as resolution, improvement, no change, or worsening during the first 24 h post-DDAVP administration based on EHR description of bleeding. Procedural bleeding was defined as greater than expected bleeding during the first 24 h post-DDAVP administration and deemed nonanatomic in nature based on EHR documentation. Outcomes after 24 h or following subsequent DDAVP doses related to the same bleeding event or invasive procedure were not recorded due to the frequent institutional use of DDAVP outside of established guidelines and potential for confounding under these circumstances [8]. DDAVP related adverse effects, and type, based on EHR documentation, were collected. Means and standard deviations for continuous variables and frequencies and percentages for categorical variables were used to summarize the data. Continuous variables were compared using a Student's *t*-test and categorical variables were compared using chi-square or Fisher's exact tests.

**Table 1:** Patient characteristics.

Characteristic	Non-ADJ group (*n* = 75)	ADJ group (*n* = 21)	*P* value
Age (years), mean (SD)	47.0 (19.2)	47.2 (17.3)	0.97
Sex, *n* (%)			
Female	66 (0.88)	20 (0.81)	0.47
Male	9 (0.12)	1 (0.19)	
Race, *n* (%)			
White	70 (0.93)	20 (0.95)	1.00
Black	4 (0.05)	1 (0.05)	
Unknown	1 (0.01)	0 (0)	
Ethnicity, *n* (%)			
Non-Hispanic or Latino	74 (0.99)	21 (1.00)	0.60
Unknown	1 (0.01)	0 (0)	
Weight (kg), mean (SD)	88.4 (20.1)	108.8 (18.1)	<0.001
BMI (kg/m^2^), mean (SD)	32.3 (7.7)	38.8 (6.8)	<0.001
^a^BMI category, *n* (%)			
Normal	56 (0.75)	0 (0)	0.01
Overweight/obese	19 (0.25)	21 (1.00)	
^b^Expected DDAVP dose (mcg), mean (SD)	26.5 (6.0)	32.6 (5.4)	<0.001
Actual DDAVP dose (mcg), mean (SD)	26.6 (6.5)	23.5 (4.9)	0.043
Actual DDAVP dose (mcg/kg), mean (SD)	0.30 (0.03)	0.22 (0.04)	<0.001
Medical condition affecting bleeding risk, *n* (%)			
^c^Chronic liver disease	1 (0.01)	0 (0)	1.00
^c^Chronic kidney disease	2 (0.03)	0 (0)	1.00
^c^Hypertension	26 (0.35)	5 (0.24)	0.43
^c^Malignancy	3 (0.04)	1 (0.05)	1.00
Stroke	1 (0.01)	0 (0)	1.00
^d^Anemia	17 (0.25)	6 (0.33)	0.70
^e^Thrombocytopenia	2 (0.03)	0 (0)	0.83
^c^ICU	4 (0.05)	1 (0.05)	1.00
^f^Antiplatelets	3 (0.04)	2 (0.10)	0.30
^f^Anticoagulants	1 (0.01)	0 (0)	1.00

ADJ, adjusted weight-based dosing; BMI, body mass index; DDAVP, desmopressin (1-deamino-8-d-arginine vasopressin).

a Defined as a BMI greater than or equal to 25 kg/m^2^.

b Calculated as 0.3 mcg/kg of actual body weight.

c Active or present at the time of bleeding or invasive procedure.

d Defined as hemoglobin less than 12 g/dl within 30 days of bleeding or invasive procedure.

e Defined as platelet count less than 150 000/ul within 30 days of bleeding or invasive procedure.

f Use at the time of or during the first 24 h post-DDAVP administration.

## Discussion

DDAVP was administered for five acute bleeds and 91 invasive procedures. Of these, the DDAVP dose was adjusted for one bleed and 20 invasive procedures. The most frequently adjusted doses were 30 and 20 mcg, 33.3 and 28.6%, respectively. The mean age was similar between the ADJ and non-ADJ dosing groups, 47.2 (SD 17.3) and 47.0 (SD 19.2) years, respectively, *P* = 0.97. Most patients were female, 81 and 88% in the ADJ and non-ADJ dosing groups, respectively, and there were no differences in sex between the two groups, *P* = 0.47. The mean weight, 108.8 vs. 88.8 kg, and BMI, 38.8 vs. 32.3 kg/m^2^, *P* < 0.001, were higher in the ADJ dosing group. All patients in the ADJ dosing group were overweight/obese compared with 25% of patients in the non-ADJ dosing group, *P* = 0.01. While the expected DDAVP dose based on ABW was higher in the ADJ dosing group, 32.6 vs. 26.6 mcg, *P* = 0.01, the actual dose of DDAVP given was lower, 23.5 vs. 26.6 mcg, *P* = 0.04. There were no differences in medical conditions affecting bleeding risk between the two groups (Table 1).

Periprocedural bleeding occurred in only 2 of 91 invasive procedures: a cesarean section complicated by postpartum hemorrhage requiring red blood cell transfusion (non-ADJ dosing group) and a sigmoid colectomy complicated by intraoperative bleeding requiring surgical packing and red blood cell transfusion (ADJ dosing group). There was no difference in periprocedural bleeding between the ADJ and non-ADJ dosing groups, 4.8 vs. 1.3%, *P* = 0.33. The most common invasive procedure was childbirth, 19.8%. Other common invasive procedures included total hip/knee arthroplasty, 13.2%; endoscopy (esophagogastroduodenoscopy (EGD), colonoscopy, and endoscopic retrograde cholangiopancreatography (ERCP)) with biopsy/polypectomy, 8.8%; spine surgery, 7.7%; and laparoscopic cholecystectomy, 7.7% (Table 2). Acute bleeds were rare and consisted of gastrointestinal bleeding, hemoperitoneum, skin laceration, soft tissue hematoma, and vaginal bleeding. DDAVP resulted in resolution of all bleeds except vaginal bleeding where improvement was noted. ADJ dosing of DDAVP was only performed with the skin laceration. One adverse effect, hyponatremia, occurred in a patient in the non-ADJ dosing group who had a BMI of 29.5 and received 24 mcg of DDAVP.

**Table 2:** ^a^Invasive procedures.

Type	Number
Childbirth	18
Cesarean section	6
Vaginal delivery	12
Joint arthroplasty	12
Total hip arthroplasty	6
Total knee arthroplasty	6
Endoscopy	8
EGD (biopsy)	4
ERCP (sphincterotomy)	2
Colonoscopy (polypectomy and biopsy)	2
Laparoscopic cholecystectomy	7
Spine surgery	7
Port placement	4
Arthroscopic joint surgery	3
Colectomy	3
Laparoscopic	1
Open	2
Incision and drainage	3
Laparoscopic bariatric	3
Laparoscopic salpingoopherectomy	3
Laparoscopic total abdominal hysterectomy	3
Thyroid surgery	3
Dental extraction	2

a Invasive procedures performed more than once.

In our study, we found no difference in periprocedural bleeding between ADJ and non-ADJ dosing of DDAVP in patients with type 1 VWD. Due to the rarity of acute bleeding events, comparison of treatment efficacy between ADJ and non-ADJ dosing of DDAVP was not feasible; however, among the five episodes, bleeding resolved/improved in all instances. The most frequently adjusted DDAVP doses were 30 and 20 mcg, which is consistent with the common practice of capping the maximum dose of DDAVP at these amounts. Only one patient, in the non-ADJ dosing group, experienced a DDAVP-related adverse effect, hyponatremia.

There is little published literature regarding this topic. One retrospective study compared patients receiving adjusted weight-based DDAVP with those receiving ABW-based DDAVP. One hundred and nine patients were included in the study and 24% of patients received adjusted weight-based DDAVP. Thirty five percentage of patients receiving adjusted weight-based DDAVP, and 37% of patients receiving ABW-based DDAVP were obese. Eighty one percentage of patients received DDAVP for bleeding and 15% of patients received DDAVP prior to an invasive procedure. There was no difference in packed red blood cell transfusion requirements, need for procedural hemostasis, or death between the two groups. Notably, patients with an underlying bleeding disorder, such as VWD, were excluded from the analysis; thus, it is unclear if these results are applicable to such patients [6].

A few weaknesses of our study warrant discussion. First, due to inherent limitations as a result of the retrospective nature of the study, it is conceivable that periprocedural bleeding or DDAVP-related adverse effects may have occurred but were not communicated to the healthcare provider or not documented in the EMR. Second, as a result of the frequent institutional use of DDAVP outside of established guidelines (i.e. only one dose of DDAVP given prior to major orthopedic surgery), outcomes were restricted to 24 h post procedure and DDAVP administration; thus, limiting the ability to draw conclusions beyond the immediate post procedure period. Third, the diagnosis of VWD was based on ICD-10 codes, and historical clinical and laboratory data were often not available to confirm the diagnosis, which can be challenging; thus, it is possible some individuals may have been incorrectly diagnosed with VWD [8].

In conclusion, we describe the safe and effective use of adjusted weight-based DDAVP immediate post procedure in patients with type 1 VWD. Future prospective studies are warranted to confirm and expand on these findings and facilitate evidence evidence-based clinical recommendations.

## Acknowledgements

Authors’ contributions: CDS designed and performed the research, completed the statistical analysis, formulated the conclusions, and wrote the paper.

Data statement: Data that support the findings of this study are available from the corresponding author upon reasonable request.

Funding source: None.

### Conflicts of interest

C.D.S. has acted as a paid consultant for Genentech, Sanofi, Novo Nordisk, and Takeda Pharmaceuticals.
